# *traG* Gene Is Conserved across* Mesorhizobium* spp. Able to Nodulate the Same Host Plant and Expressed in Response to Root Exudates

**DOI:** 10.1155/2019/3715271

**Published:** 2019-01-30

**Authors:** A. Paço, J. R. da-Silva, F. Eliziário, C. Brígido, S. Oliveira, A. Alexandre

**Affiliations:** Laboratório de Microbiologia do Solo, Instituto de Ciências Agrárias e Ambientais Mediterrânicas (ICAAM), Instituto de Investigação e Formação Avançada (IIFA), Universidade de Évora, Apartado 94, 7002-554 Évora, Portugal

## Abstract

Evidences for an involvement of the bacterial type IV secretion system (T4SS) in the symbiotic relationship between rhizobia and legumes have been pointed out by several recent studies. However, information regarding this secretion system in* Mesorhizobium *is still very scarce. The aim of the present study was to investigate the phylogeny and expression of the* traG* gene, which encodes a substrate receptor of the T4SS. In addition, the occurrence and genomic context of this and other T4SS genes, namely, genes from* tra/trb *and* virB/virD4* complexes, were also analyzed in order to unveil the structural and functional organization of T4SS in mesorhizobia. The location of the T4SS genes in the symbiotic region of the analyzed rhizobial genomes, along with the* traG* phylogeny, suggests that T4SS genes could be horizontally transferred together with the symbiosis genes. Regarding the T4SS structural organization in* Mesorhizobium*, the* virB/virD4* genes were absent in all chickpea (*Cicer arietinum* L.) microsymbionts and in the* Lotus* symbiont* Mesorhizobium japonicum* MAFF303099^T^. Interestingly, the presence of genes belonging to another secretion system (T3SS) was restricted to these strains lacking the* virB/virD4* genes. The* traG* gene expression was detected in* M. mediterraneum* Ca36^T^ and* M. ciceri* LMS-1 strains when exposed to chickpea root exudates and also in the early nodules formed by* M. mediterraneum* Ca36^T^, but not in older nodules. This study contributes to a better understanding of the importance of T4SS in mutualistic symbiotic bacteria.

## 1. Introduction

Rhizobia are able to fix atmospheric nitrogen when in symbiosis with legumes, providing ammonia to these plants. The rhizobial symbiosis genes include two main classes of genes, namely, nodulation and nitrogen fixation genes [1]. Nodulation genes (e.g.,* nodABC*) are implicated in biosynthesis and secretion of Nod factors, molecules involved in root infection, and nodule development. Nitrogen fixation genes include genes involved in the synthesis, processing, and assembly of nitrogenase complex (e.g.,* nifHDK, fixGH*), responsible for N_2_-fixation [2–4]. The expression of these sets of genes is regulated by two key transcriptional regulators, namely, NodD for nodulation genes and NifA for nitrogen fixation genes [3, 5]. Nevertheless, the involvement of other bacterial genes in legume-rhizobia symbioses has been described. Some studies have shown that genes commonly found among prokaryotes and involved in a diversity of cellular mechanisms have also a role in legume-*Rhizobium* symbiosis, as, for example, stress response genes [6–8], quorum sensing, or secretion system genes [9–12].

Although several studies have shown the involvement of the rhizobial secretion systems in the symbiotic rhizobia-legume relationship [11–13], their precise role is far from being fully understood. Such transfer systems are ancestrally related to virulence (transmission of antibiotic resistance genes and virulence genes) and to the mating pair formation complexes in pathogenic bacteria [14]. However, with the increasing number of available rhizobia genomes, the occurrence of these systems has also been described in symbiotic bacteria [12]. These systems allow DNA uptake or release, i.e., the translocation of genetic material between bacterial strains or between bacteria and eukaryotic cells [15, 16]. In addition, types III (T3SS), IV (T4SS), and VI (T6SS) systems have the ability to directly translocate bacterial proteins into the cells of eukaryotic susceptible hosts [17, 18]. Therefore, several studies suggest a role for this translocated bacterial material in the primary interplay between bacteria and their hosts for the establishment of a mutualistic symbiosis [10, 13].

In rhizobia, the studies regarding the T4SS are restricted to* Mesorhizobium japonicum* [19, 20],* Rhizobium etli* [16],* Ensifer meliloti,* and* Ensifer medicae *[12, 21]. However, it is considered that, in general, the rhizobial T4SS protein apparatus is similar to the bacterial pathogen* Agrobacterium tumefaciens*. In the* A. tumefaciens* strain C58 three types of T4SS genes were described:* vir*,* avh*, and* trb* [21]. The Tra/Trb and VirB/VirD4 protein complexes are the most studied. The first one is responsible for the conjugative transfer of the Ti plasmid between bacteria and the second one for the delivery T-DNA and effectors proteins to plant cells [22]. In these complexes, the TraG and VirD4 proteins (coupling proteins) have a role as substrate receptors, corresponding to the first T4SS components that contact the substrate (effector proteins or DNA), recruiting specific substrates to the translocation channel [17, 18, 22, 23]. On the other hand, the Trb and VirB proteins are involved in the assembly of the translocation channels [22].

One of the evidences that points out the symbiotic importance of T4SS is the genomic location of the T4SS genes. In* M. japonicum* R7A strain, the genes coding for the Tra/Trb and VirB/VirD4 complexes were found to be located in the symbiosis island [20]. Besides that, the expression of T4SS* virB/virD4* genes seems to be regulated by NodD, which is the main regulator of* nod* genes expression [19, 21]. Therefore, the T4SS* virB/virD4* genes expression could be temporally coordinated with Nod Factors production, suggesting a function of T4SS VirB/VirD4 proteins in the early steps of the legume-rhizobia symbioses [9, 11, 20]. Nevertheless, this finding of coordinated expression between T4SS and* nod* genes [20] did not include the analysis of genes coding for the T4SS Tra/Trb complex. Up to now, it is unknown if any relation exists between the expression of* tra/trb* genes and some crucial step for the establishment of symbiotic rhizobia-legume relationship.

In addition to the findings mentioned above, an interesting aspect observed in some* Lotus *symbionts is that strains harboring the T4SS* virB/virD4* genes do not present T3SS genes and* vice versa *[19]. This suggests that the symbiotic role of T4SS could be analogous to the symbiotic role of T3SS, even more because in some rhizobia the expression of T3SS genes is also regulated by NodD, and this secretion system is located in the symbiotic island [9].

The present study aimed to contribute to our understanding of the structural and functional organization of T4SS in mesorhizobia, using mesorhizobia strains able to nodulate chickpea (*Cicer arietinum *L.) as a study case. The phylogeny and expression of the T4SS* traG* gene, which belongs to the T4SS* tra/trb* complex, were investigated. Moreover, the occurrence and genomic context of this and other T4SS genes from the* tra/trb* and* virB/virD4* complexes were also analyzed in several mesorhizobia strains. Interestingly, the T4SS* tra/trb* complex is present in all the studied* Mesorhizobium* strains, contrarily to the T4SS* virB/virD4* complex, which was not detected in the* Cicer arietinum *mesorhizobia analyzed.

## 2. Materials and Methods

### 2.1. Bacterial Strains, Growth Conditions, and DNA Extraction

The bacterial strains used in this work are listed in Table 1. From the 35 rhizobia strains analysed, 21 are* Cicer arietinum* symbionts and, from these, 16 are native isolates obtained from a collection of Portuguese* Cicer arietinum* mesorhizobia previously characterized [25–27]. The remaining 14 rhizobia strains are able to nodulate a diverse range of plant hosts (Table 1).

The total DNA of 18* Cicer arietinum* mesorhizobia strains grown in TY medium during 16 hours at 28°C was extracted using the E.Z.N.A. bacterial DNA kit (Omega Bio-Tek), according to the manufacturer's instructions.

### 2.2. Amplification of* traG, nod,* and* nifA* Genes

The* traG* gene was amplified using the primers traGF 5′-ATGCTGACCTACCAGACGCC-3′ and traGRint 5′-CGGAAACTCGTCCAGCATCA-3′, designed to target conserved regions of this gene in* Mesorhizobium*, which allow the amplification of an internal region of ~ 1155 bp. The* nodD* and* nifA *genes were amplified using primers NodDF 5′-ATGCGTTTCAAAGGACTTG-3′, NodDR 5′-TCACAGCGGGGCAGCCATCC-3′, NifAF 5′-ATGGGCTGCCAAATGGAACG-3′, and NifAR 5′-TCAGAGACGCTTGATCTCGA-3′. These primers allow the amplification of nearly complete sequences of these two genes (918 bp for* nodD* and 1059 bp for* nifA*,). The PCR reactions were performed in a final volume of 50 *μ*L, using 20 ng of total DNA, 1× reaction buffer, 0.2 mM of each dNTP, 1.5mM of MgSO_4 _(*traG *and* nifA*) or 1 mM of MgSO_4_ (*nodD*), 15 pmol of each primer, and 0.02 U/*μ*L of KOD Hot Start DNA polymerase (Merck Millipore). The amplification programs were 2 min of initial denaturation at 95°C and 30 cycles of 20 s at 95°C, followed by 10 s of annealing at 58°C (*traG* and* nodD*) or 62°C (*nifA*) and an extension step of 15 s (*nodD*), 17 s (*nifA*), or 23 s (*traG*) at 70°C. PCR products were purified using the GFX DNA purification kit (GE Healthcare) or MinElute Gel Extraction kit (QIAGEN) following the manufacturer's instructions. Sequencing reactions were performed by Macrogen Europe (Amsterdam, Netherlands).

The sequences of* nodD, nifA,* and* traG *genes from the* Cicer arietinum* mesorhizobia strains have been deposited in the GenBank database under the accession numbers KT966793 to KT966810, KT966811 to KT966828 and KT966829 to KT966846, respectively (Table 1).

### 2.3. Phylogenetic Analysis

The* nodD*,* nifA*,* traG,* and 16S rRNA nucleotide sequences from several rhizobia strains able to nodulate different plant hosts (*Anthyllis vulneraria*,* Biserrula pelecinus*,* Bituminaria bituminosa*,* Cicer arietinum*,* Glycine max*,* Lotononis carinata*,* Lotus *spp., and* Medicago sativa*) were either obtained in this study or retrieved from NCBI or JGI IMG database [24, 28] (Table 1). Sequences were analyzed and aligned using BIOEDIT (version 7.0.4.1) [29]. Molecular phylogenies for* nodD*,* nifA*,* traG,* and 16S rRNA nucleotide sequences were generated with MEGA6 version 6.0.6 [30], using the Maximum Likelihood method, with the distance correction calculated by Tamura 3-parameter model, with rate among sites gamma distributed for* nodD* phylogeny and rate among sites gamma distributed with invariant sites for* nifA*,* traG,* and 16S rRNA gene phylogenies. The phylogenetic trees were rooted using the* Bradyrhizobium elkanii* and* E. meliloti* species as outgroup. Robustness of tree nodes was evaluated using bootstrap analyses, with 1000 replicates.

### 2.4. Analysis of the Genomic Regions Containing the T4SS Genes, Symbiosis Genes, and T3SS Genes

The occurrence and genome context of T4SS genes, symbiosis genes, and two T3SS genes were analyzed in the genomes of* Cicer arietinum* symbionts (*Mesorhizobium mediterraneum* UPM-Ca36^T^,* M. muleiense *CGMCC 1.11022^T^*, M. ciceri* LMS-1,* M. ciceri* CMG6, and* M. ciceri* CC1192),* Biserrula pelecinus* symbionts (*M. ciceri* bv.* biserrulae* WSM1271,* M. australicum* WSM2073^T^, and* M. opportunistum* WSM2075^T^), and* Lotus* spp. symbionts (*M. loti* NZP2037,* M. japonicum* R7A, and* M. japonicum* MAFF303099^T^) (former* M. loti* strains, reclassification according to [31]) using local BLAST tool from BIOEDIT (version 7.0.4.1) [29]. All these genomes are available in JGI IMG or NCBI genome database. The* M. ciceri* LMS-1 genome sequencing data were obtained in our lab (Bioproject accession PRJNA507072) and the contigs used in this work were submitted to NCBI (accession numbers MK226192 to MK226197).

In total, the localization of 38 genes was analyzed, namely,* nod* genes (*nodA*,* nodB*,* nodC*, and* nodD*),* nif* genes (*nifA*,* nifH*,* nifD*,* nifK*,* nifE*, and* nifN*),* fix* genes (*fixG*,* fixH*),* vir* genes (*virA*,* virG*,* virB1*,* virB2*,* virB3*,* virB4*,* virB5*,* virB6*,* virB7*,* virB8*,* virB9*,* virB10*,* virB11*, and* virD4*),* tra/trb* genes (*traG*,* trbB*,* trbC*,* trbD*,* trbE*,* trbJ*,* trbL*,* trbF*,* trbG,* and* trbI*), and the T3SS genes* rhc*J and* rhc*N (named as in [32]). This analysis was based on the comparison of these mesorhizobial genomes with the well-characterized symbiosis island of* M. japonicum* R7A, in which the T4SS genes are located [33].

### 2.5. Analysis of* traG *and* nodA *Genes Expression in* M. ciceri* LMS-1 and* M. mediterraneum* UPM-Ca36^T^

To evaluate the* traG* and* nodA* genes expression in the* Cicer arietinum*-nodulating* M. mediterraneum* UPM-Ca36^T^ and* M. ciceri* LMS-1, the total RNA from those strains was extracted from free-living cell cultures with and without exposure to* Cicer arietinum* root exudates and also from the bacteroids at two different time points.

The root exudates were obtained as described by [34], with slight modifications, namely using minimal medium described by [35]. For expression analysis in free-living conditions, with and without root exudates, cell cultures were grown in five mL of liquid TY medium at 28°C until exponential-phase. After centrifugation at 8000 g during five minutes, cells were resuspended in five mL of root exudates and incubated for 24 hours at 28°C. Five mL of minimal medium [35] was used to resuspend cells not exposed to root exudates and these were incubated under the same conditions as previously mentioned.

For expression analysis in bacteroids,* Cicer arietinum* plants were grown and inoculated as described by [36], being then used to collect root nodules at 15 and 25 days after rhizobial inoculation (dpi). The nodules were treated for posterior RNA extraction as described by [37]. Total RNA of free-living bacteria and bacteroids was extracted using the GeneJET™ RNA Purification Kit (ThermoFisher Scientific). DNA contamination was removed by digestion with DNase I (Roche Diagnostics), followed by RNA cleanup using the same RNA Purification Kit mentioned before. Approximately, 1 *μ*g of total RNA was subjected to reverse transcription for cDNA synthesis, using the RevertAid First Strand cDNA Synthesis kit (ThermoFisher Scientific).

The* traG* and* nodA* genes expression was analyzed by semiquantitative RT-PCR analyses as described in [38]. The cDNA previously obtained was used for PCR amplification of partial sequences of the* traG* and* nodA *genes (primers traGIntF 5′- GGCCAATCTACAAGCCGTGG -3′ and traGIntR3 5′- GCCCACCGTGAAGACCCATA -3′ for* traG*; primers NodAIntF 5′- ccgaatgtcgagtggaagtt -3′ and NodAIntR3 5′- ctcgccaactttgatgaagc -3′ for* nodA*), which generates a fragment of 193 and 234 bp, respectively. These PCR reactions were performed in a final volume of 50 *μ*L, using 2 *μ*L of cDNA (~40 ng), 1× reaction Green GoTaq® Flexi buffer, 0.2 mM of each dNTP, 1.5 mM MgCl_2, _15 pmol of each primer and 0.025U/*μ*L of GoTaq® G2 Flexi DNA Polymerase (Promega, Fitchburg, U.S.A). The amplification program was 2 min of initial denaturation at 95°C, 30 cycles of 60 s at 95°C, 60 s at 59°C (*traG*) or 54°C (*nodA*), 12 s (*traG*), or 14 s (*nodA*) at 72°C and a final extension of 5 min at 72°C.

The amplification of the 16S rRNA gene was used to normalize the relative* traG *and* nodA *transcript abundance. Primers IntF and IntR [25] were used to generate a fragment of 199 bp. This PCR reaction was performed in a final volume of 50 *μ*L, using 2 *μ*L of cDNA (~40 ng), 1× reaction Green GoTaq® Flexi buffer, 0.2 mM of each dNTP, 1.5 mM MgCl_2_, 15 pmol of each primer, and 0.025U/*μ*L of GoTaq® G2 Flexi DNA Polymerase (Promega, Fitchburg, USA). The amplification program was 2 min of initial denaturation at 95°C, 30 cycles of 60 s at 95°C, 60 s at 56°C, 12 s at 72°C, and a final extension of 5 min at 72°C. Densitometric analyses of ethidium bromide-stained agarose gels were performed using Kodak Digital Science 1D version 2.0.3 (Eastman Kodak Company). Positive controls with total DNA of* M. ciceri* LMS-1 and* M. mediterraneum* UPM-Ca36^T^ as template and negative controls without reverse transcriptase enzyme were performed. Three biological replicates were used for the expression analysis of the genes mentioned above.

The data obtained from the RT-PCR analyses were compared using Student's* t*-test (differences were considered statistically significant at* P*<0.05, representing the 95% confidence interval).

## 3. Results

### 3.1. Phylogenetic Analysis

**Figure 1:**
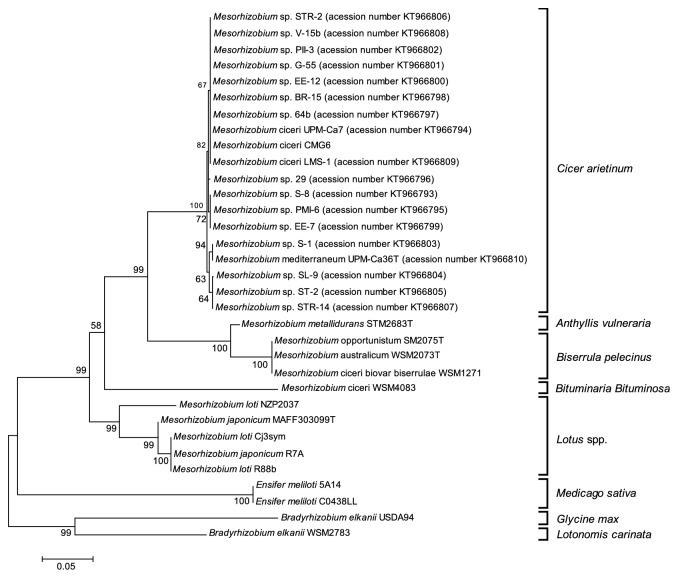
Maximum Likelihood tree based on* nodD *gene sequences (alignment length of 731 bp). Tamura 3-parameter model with rate among sites gammas distributed was used. Percentage of bootstrap support (1000 replicates) is indicated on internal branches. Scale bar indicates 0.05 substitutions* per site*.

**Figure 2:**
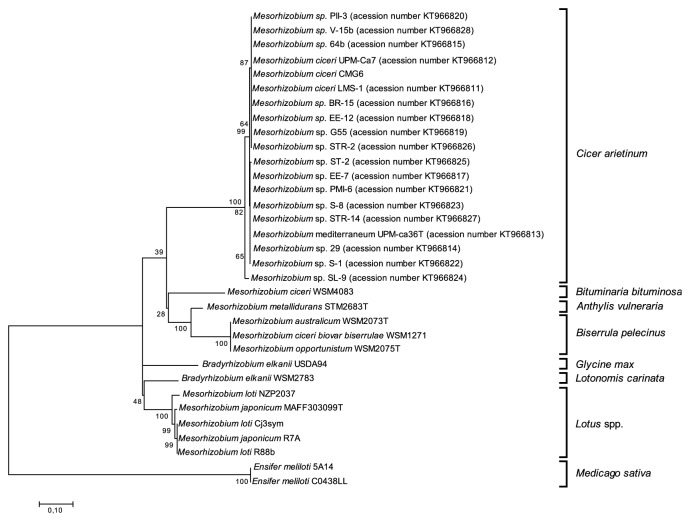
Maximum Likelihood tree based on* nifA *gene sequences (alignment length of 1014 bp). Tamura 3-parameter model with rate among sites gamma distributed with invariant sites was used. Percentage of bootstrap support (1000 replicates) is indicated on internal branches. Scale bar indicates 0.1 substitutions* per site*.

**Figure 3:**
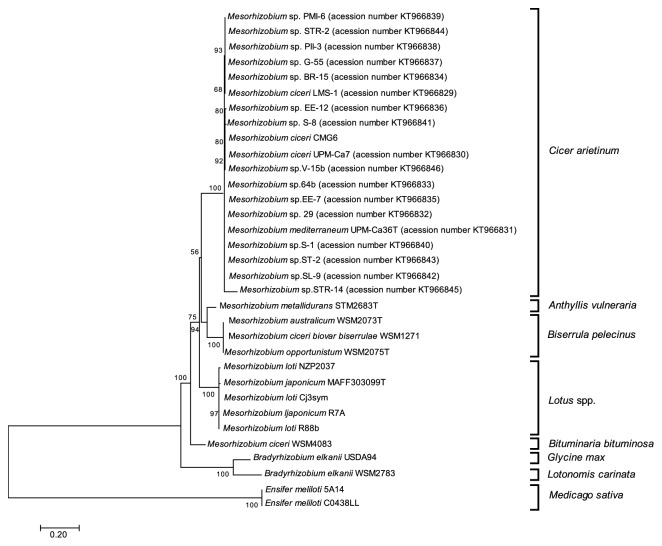
Maximum Likelihood tree based on* traG *gene sequences (alignment length of 1004 bp). Tamura 3-parameter model with rate among sites gamma distributed with invariant sites was used. Percentage of bootstrap support (1000 replicates) is indicated on internal branches. Scale bar indicates 0.2 substitutions* per site*.

**Figure 4:**
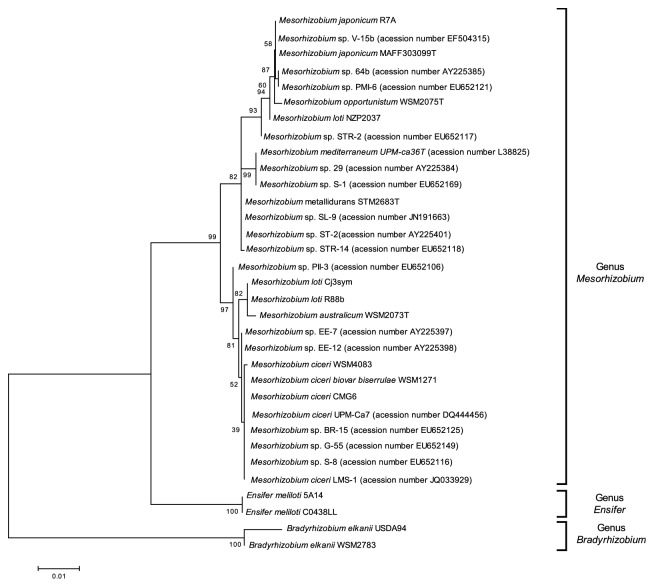
Maximum Likelihood tree based on 16S rRNA gene sequences (alignment length of 1278 bp). Tamura 3-parameter model with rate among sites gamma distributed with invariant sites was used. Percentage of bootstrap support (1000 replicates) is indicated on internal branches. Scale bar indicates 0.01 substitutions* per site*.

With the purpose of comparing the phylogeny of the* traG* gene with that of symbiosis genes, the* nodD, nifA,* and* traG *nucleotide sequences of these genes were analyzed for 33 mesorhizobia strains able to nodulate different plant hosts (*Anthyllis vulneraria, Biserrula pelecinus, Bituminaria bituminosa, Cicer arietinum, Glycine max, Lotononis carinata, Lotus *spp*., *and* Medicago sativa*) (Figures 1, 2, and 3, respectively). In order to also compare the* traG* phylogeny with the species tree, a phylogeny based on the taxonomic marker 16S rRNA gene was also generated using the same set of strains (Figure 4).

As expected, the phylogenetic trees based on sequences of the symbiosis genes* nodD* (Figure 1) and* nifA* (Figure 2) showed similar topologies. Well defined clusters that correspond to the different host plants were identified, reflecting the high level of sequence conservation among strains that nodulate the same host legume. Three main clusters of mesorhizobia strains could be distinguished: mesorhizobia able to nodulate* Cicer arietinum*; strains that were isolated from* Biserrula pelecinus* grouping closer with a strain nodulating* Anthyllis vulneraria*; and mesorhizobia able to nodulate several* Lotus *species.* M. ciceri* WSM 4083, a symbiont of* Bituminaria bituminosa,* grouped apart from these clusters (Figures 1 and 2). Contrary to the* nodD* phylogenetic analysis, the* nifA* phylogeny reflects the fact that* Lotus *symbionts share a higher* nifA* sequence similarity with* Bradyrhizobium *strains than with other mesorhizobia, which nodulate* Cicer arietinum *and* Biserrula pelecinus* (Figure 2).

Similarly to the phylogenies based on symbiosis genes (*nodD* and* nifA*), the* traG* based-phylogeny also showed rhizobia strains clustering according to their host range (Figure 3), rather than species affiliation. For instance, all symbionts of* Cicer arietinum, Biserrula pelecinus,* or* Lotus *spp. formed separated clusters. The* traG* gene is conserved among the* Mesorhizobium* species able to nodulate the same host plant, which suggests that this gene was prone to horizontal gene transfer events.

As expected, the 16S rRNA-based phylogeny presented a different topology from those based on the* nodD* or* nifA* genes. This phylogeny comprises three main clusters, corresponding to different genera, namely,* Mesorhizobium*,* Ensifer, *and* Bradyrhizobium* (Figure 4).* Cicer arietinum* mesorhizobia native isolates are found scattered along the* Mesorhizobium* cluster.

### 3.2. Genomic Localization and Organization of T4SS Genes, T3SS Genes, and Symbiosis Genes

**Figure 5:**
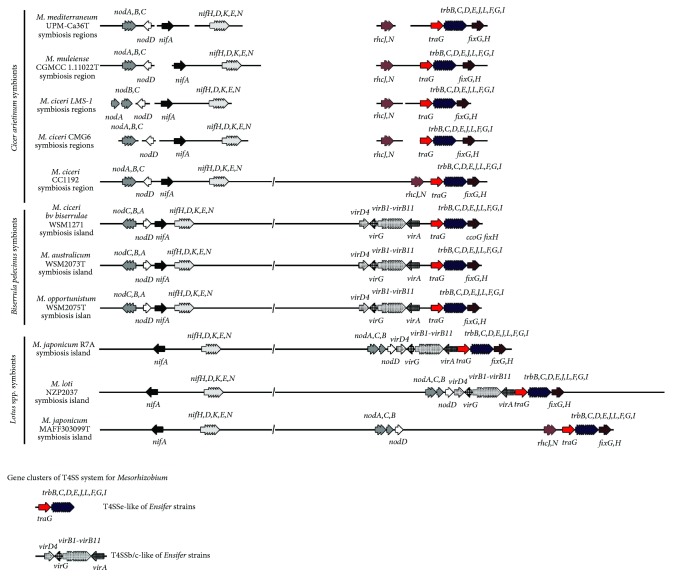
Genomic context analysis of T4SS, T3SS and symbiosis genes in the symbiotic islands/regions of the genomes of several mesorhizobia strains. This analysis includes the genomes of symbionts of three different host plants, namely* Cicer arietinum*,* Biserrula pelecinus,* and different* Lotus *species. The* tra/trb *and* virB/virD4* complexes identified in the mesorhizobia strains and studied in this work present similarities with the T4SSe-like and T4SSb/c-like of* Ensifer* strains, according to [21]. Slash sign (/) indicates a random genetic distance.

*In silico* analyses for genomic localization of the T4SS genes (both the* tra/trb* and* virB/virD4* complexes), symbiosis genes, and two T3SS genes were performed using genomic data from several mesorhizobia strains (Figure 5). Symbionts of* Cicer arietinum, Biserrula pelecinus,* and* Lotus *spp. were included in these analyses. Nevertheless, we need to consider that the genomes of majority of the* Cicer arietinum* symbionts analyzed are in draft* status*, with the exception of the* M. ciceri* CC1192 genome, which is complete [39].

In all the genomes analyzed, the T4SS* traG/trb* genes complex was always found near nitrogen fixation genes, namely,* fixG* and* fixH *(Figure 5). In addition, BLAST analyses suggest that none of the five* Cicer arietinum *microsymbionts (*M. mediterraneum *UPM-Ca36^T^,* M. muleiense* CGMCC 1.11022^T^,* M. ciceri* LMS-1,* M. ciceri *CMG6, and* M. ciceri* CC1192) include the T4SS* virB/virD4* gene complex, homologous to the* virB/virD4* of the* M. japonicum* R7A. Nevertheless, all the analyzed* Cicer arietinum *mesorhizobia strains encode genes assigned to the T3SS, namely,* rhc*J and* rhc*N, which were not found in the* M. japonicum* R7A and* M. loti* NZP2037 genomes or in the genomes of* Biserrula pelecinus* nodulating strains. For two* Cicer arietinum* mesorhizobia species the genomic data available shows the localization of these T3SS genes near the* tra/trb* complex and consequently close to the* fixG *and* fixH* genes. Similarly to the* Cicer arietinum*-nodulating mesorhizobia strains, the* M. japonicum* MAFF303099^T^, a* Lotus *spp. symbiont, does not encode the* virB/virD4* genes and also shows the T3SS* rhc*J and* rhc*N genes located close to the* tra/trb* complex (Figure 5).

Interestingly, in strains* M. japonicum *R7A and* M. loti* NZP2037, the* nod* genes are localized nearby the* virB*/*virD4 *T4SS genes and not close to the* nif* genes as in symbionts of* Biserrula pelecinus* (*M. ciceri* bv.* biserrulae* WSM1271,* M. australicum* WSM2073^T^, and* M. opportunistum* WSM2075^T^). In* M. japonicum* MAFF303099^T^, the* nod* cluster is neither close to the* nif* genes nor close to the T4SS or T3SS analysed genes. For most of* Cicer arietinum* mesorhizobia genomes analysed, the* nod* genes were detected in a different scaffold from that of* nif*, T4SS, or T3SS genes. Nevertheless, these data are consistent with an organization similar to the one found in the complete genome of* M. ciceri* CC1192, a* Cicer arietinum *symbiont (Figure 5).

### 3.3. Analyses of* traG *and* nodA *Genes Expression by Semiquantitative RT-PCR

**Figure 6:**
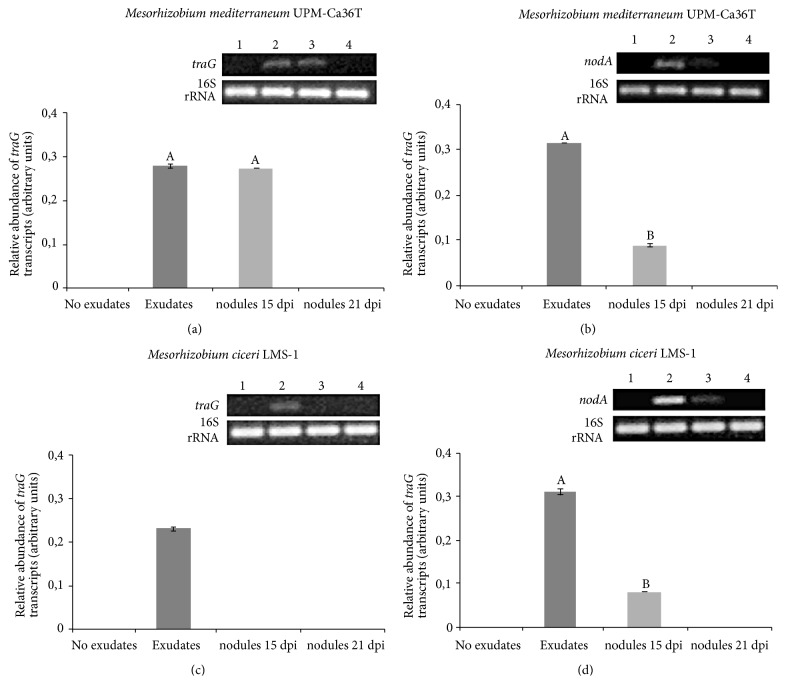
Analysis of* tra*G and* nod*A gene expression by semiquantitative RT-PCR in* M. mediterraneum* UPM-Ca36^T^ and* M. ciceri* LMS-1 strains. These genes expression level were evaluated in bacteria (1; no exudates) nonexposed to* Cicer arietinum* exudates (2; exudates) exposed to* Cicer arietinum* root exudates and in bacteroids within nodules collected (3; nodules 15 dpi) 15 days after inoculation and (4; nodules 21 dpi) bacteroids within nodules of plants collected 21 days after inoculation. (a)* M. mediterraneum* UPM-Ca36^T^* tra*G expression. (b)* M. mediterraneum* UPM-Ca36^T^* nod*A gene expression. (c)* M. ciceri* LMS-1* traG* expression. (d)* M. ciceri* LMS-1* nod*A expression. Relative abundance of* traG* and* nodA* transcripts was normalized against 16S rRNA gene expression. Data are presented as the mean and standard deviation values of three independent biological replicates. Different letters (A, B) correspond to statistical significant differences (*P < 0.05*).

To understand the timing of the T4SS* traG* gene expression, the expression of this gene and of the* nodA* gene was evaluated by semiquantitative RT-PCR in free-living bacteria grown in the presence and absence of* Cicer arietinum* root exudates and also in bacteroids from root nodules collected at two different time points (15 and 21 days after inoculation) (Figure 6). The* traG* and* nodA* gene expression analyses were performed for* M. mediterraneum *UPM-Ca36^T^ and* M. ciceri* LMS-1 strains, both* Cicer arietinum *symbionts. In free-living conditions, the* traG* gene expression was only detected when bacteria were exposed to* Cicer arietinum *root exudates (Figures 6(a) and 6(c)). In bacteroids, the* traG* gene expression was only detected in developing nodules (collected at 15 dpi) and exclusively for nodules induced by* M. mediterraneum *UPM-Ca36^T^. In older nodules (21 dpi) the* traG* transcripts were no longer detected. For the strain UPM-Ca36^T^_,_ approximately the same levels of* traG* transcripts were detected for bacteria exposed to exudates and bacteroids within 15 dpi nodules. As expected, in both strains, the* nodA* gene expression (regulated by NodD) was only observed in free-living bacteria when exposed to* Cicer arietinum* root exudates and in early stage of the nodulation process (15 dpi nodules) (Figures 6(b) and 6(d)). Nevertheless, the abundance of* nodA* transcripts in those nodules was significantly lower compared to the levels of transcription detected for this gene when free-living bacteria were exposed to* Cicer arietinum* root exudates.

## 4. Discussion

Although some studies have already analyzed rhizobia nodulating* Lotus* and* Medicago* species [12, 19–21], little is known about the T4SS in* Cicer arietinum* mesorhizobia. Herein, the mesorhizobia* traG* phylogeny and expression of this gene in* Cicer arietinum *mesorhizobia strains, as well as the occurrence and genomic context of this and other T4SS genes, were investigated.

The phylogenetic analysis performed using the T4SS* traG* gene sequences from native* Cicer arietinum* mesorhizobia isolates, together with* traG* sequences from other mesorhizobia with genomes completely or partially sequenced, shows that mesorhizobia strains nodulating the same host plant group in the same cluster, regardless of their species affiliation. The* traG*-based phylogeny is similar to the ones obtained by the phylogenetic analysis of the symbiosis genes* nodD* and* nifA *and consistent with previous studies reporting phylogenies of symbiosis genes [36, 40]. This suggests that the* traG* gene may be prone to horizontal gene transfer together with the symbiosis genes. This is further supported by the genomic context analysis performed in this work for 11 mesorhizobia, which shows proximity in terms of localization between the* traG/trb *genes and the symbiosis genes* fixG* and* fixH, *involved in bacterial nitrogen fixation [3]. Moreover, our analysis also verified that the* traG *gene is localized within the previously identified symbiosis island of* Biserrula pelecinus* and* Lotus* spp. symbionts, namely, for* M. ciceri* bv.* biserrulae* WSM1271 [41],* M. australicum* WSM2073^T^ [42],* M. opportunistum* WSM2075^T^ [43],* M. japonicum* R7A [33],* Mesorhizobium loti* NZP2037 [44], and* M. japonicum* MAFF303099^T^ [45].

Horizontal transfer of symbiosis genes between different species on the soil would allow a rhizobia strain to acquire the ability to nodulate a new host, when receiving a specific set of symbiosis genes [40, 46–52]. A well-known example of this event was reported for the strains* M. australicum *WSM2073^T^ and* M. opportunistum *WSM2075^T^, which seem to have received the complete symbiotic island from the inoculant strain* M. ciceri *bv.* biserrulae *WSM1271 and therefore gained the ability to nodulate the introduced legume* Biserrula pelecinus* [53, 54]. The present work suggests that the nonsymbiotic gene* traG* may have been transferred horizontally between strains that nodulate the same host. In fact, the TraG protein could be involved in the effective transference of the symbiotic region in these mesorhizobia strains, since this protein has been described as having a crucial function in bacterial conjugation [55] and was shown to be required for horizontal gene transfer of the symbiosis island from* Azorhizobium caulinodans* to other rhizobia [56].

Analysis of the* M. japonicum* R7A genome [33, 57] suggested at least two types of T4SS genes clusters in* Mesorhizobium* genomes, namely, the* tra/trb* and the* virB/virD4 *genes. Similar organization of these genes clusters was reported for* Ensifer* strains, namely, the T4SSb/c (*virB/virD4*) and T4SSe (*tra/trb*) clusters [21]. Our analysis shows that this is true for other mesorhizobia strains, namely,* M. ciceri bv. biserrulae* WSM1271 [40, 57],* M. australicum* WSM2073^T^ [42],* M. opportunistum* WSM2075^T^ [43], and* M. loti* NZP2037 [44, 58]. However, the work of Hubber and collaborators [19] shows that the structural organization of the T4SS in mesorhizobia is not always composed by both* tra/trb* and* virB/virD4* gene clusters. In the* Lotus* symbiont* M. japonicum* MAFF303099^T^, the absence of the T4SS* virB/virD4* complex was reported [19]. These authors proposed that in* M. japonicum* MAFF303099^T^ the absence of these genes is somehow compensated by the presence of T3SS genes, which are not encoded in the* M. japonicum* R7A genome. The present analysis of* Cicer arietinum *symbionts contributes to our understanding about this topic, supporting the idea that the absence of the T4SS* virB/virD4* gene cluster could be more common in mesorhizobia genomes than initially assumed, since in these* Cicer arietinum *mesorhizobia strains the T4SS* virB/virD4* complex seems to be absent, while the T3SS genes were detected in all the strains. Altogether, these data could also suggest that, in mesorhizobia, the role(s) of the T4SS VirB/VirD4 proteins could be at least partially substituted by the role(s) of the T3SS proteins. Further studies are required to verify this putative functional redundancy.

The analysis of the* traG* gene expression in two* Cicer arietinum *symbionts,* M. mediterraneum* UPM-Ca36^T^, and* M. ciceri* LMS-1 shows that this gene is expressed in free-living bacteria when exposed to* Cicer arietinum *root exudates and may also be expressed in recently formed root nodules. A very similar expression profile is observed for the nodulation gene* nodA*. Although these findings are consistent with a putative regulation of the* traG* gene by NodD, which would be similar to what was previously reported for T4SS* virB/virD4 *genes as well as T3SS genes [21], no* nod*-box was detected upstream the* traG* gene in the mesorhizobia genomes analyzed. The expression of* traG* during nodulation was also supported by another study reporting high-resolution transcriptome analyses in bacteroids of* Ensifer *sp. NGR234, which detected T4SS gene expression (at very low levels), in* Vigna unguiculata* and* Leucaena leucocephala* nodules [59]. Ling and collaborators [56], using a gene knockout approach, showed that TraG is required for conjugative DNA transfer in rhizobia. A role of TraG in horizontal transfer events of symbiosis islands is consistent with the timing of expression detected for this gene, which in this work was seen to be activated by legume exudates and even in developing nodules.

## 5. Conclusions

The location of T4SS genes clusters in the symbiotic regions of rhizobia genomes, as well as the timing of the* traG* gene expression, supports the previously reported role of TraG in the horizontal transfer events of symbiosis genes. Moreover, the phylogeny of* traG* is similar to the* nodD* and* nifA* phylogenies, which suggests that* traG *is horizontally transferred together with these symbiosis genes. This study addressing several aspects of the* traG* gene in mesorhizobia contributes to a better understanding of the structural and functional organization of this secretion system in mutualistic symbiotic bacteria.

## Figures and Tables

**Table 1:** Rhizobia strains used in the present study.

**Species**	**Strains/** **Isolates**	**Host plant**	** NCBI Accession number**				**JGI Bioproject ID ** **NCBI Genome**
			***nodD***	***nifA***	***traG***	**16S rRNA**	
*Bradyrhizobium elkanii*	WSM2783	*Lotononis carinata*					PRJNA163061
*Bradyrhizobium elkanii*	USDA94	*Glycine max*					PRJNA165317
*Ensifer meliloti*	5A14	*Medicago sativa*					PRJNA167593
*Ensifer meliloti*	C0438LL	*Medicago sativa*					PRJNA47287
*Mesorhizobium australicum*	WSM2073^T^	*Biserrula pelecinus*					PRJNA47287
*Mesorhizobium ciceri bv. biserrulae*	WSM1271	*Biserrula pelecinus*					PRJNA48991
*Mesorhizobium ciceri *	CMG6	*Cicer arietinum*					PRJNA182744
*Mesorhizobium ciceri*	UPM-Ca7	*Cicer arietinum*	** KT966794**	** KT966812**	**KT966830**	DQ444456	NA
*Mesorhizobium ciceri*	WSM4083	*Bituminaria bituminosa*					PRJNA78191
*Mesorhizobium ciceri *	LMS-1	*Cicer arietinum*	** KT966809**	** KT966811**	**KT966829**	JQ033929	PRJNA507072
*Mesorhizobium ciceri*	CC1192	*Cicer arietinum*					PRJNA317272
*Mesorhizobium japonicum*	MAFF303099^T^	*Lotus *spp.					PRJNA18
*Mesorhizobium japonicum*	R7A	*Lotus *spp.					PRJNA74389
*Mesorhizobium loti*	CJ3sym	*Lotus *spp.					PRJNA165305
*Mesorhizobium loti*	NZP2037	*Lotus *spp.					PRJNA81803
*Mesorhizobium loti*	R88b	*Lotus *spp.					PRJNA76961
*Mesorhizobium mediterraneum*	UPM-Ca36^T^	*Cicer arietinum*	** KT966810**	** KT966813**	**KT966831**	L38825	NZ_NPKI00000000
*Mesorhizobium metallidurans*	STM 2683^T^	*Anthyllis vulneraria*					PRJEB1501
*Mesorhizobium muleiense*	CGMCC 1.11022^T^	*Cicer arietinum*					PRJNA329780
*Mesorhizobium opportunistum*	WSM2075^T^	*Biserrula pelecinus*					PRJNA33861
*Mesorhizobium *sp.	29	*Cicer arietinum*	** KT966796**	** KT966814**	**KT966832**	AY225384	NA
*Mesorhizobium *sp.	64b	*Cicer arietinum*	** KT966797**	** KT966815**	**KT966833**	AY225385	NA
*Mesorhizobium *sp.	BR-15	*Cicer arietinum*	** KT966798**	** KT966816**	**KT966834**	EU652125	NA
*Mesorhizobium *sp.	EE-7	*Cicer arietinum*	** KT966799**	** KT966817**	**KT966835**	AY225397	NA
*Mesorhizobium *sp.	EE-12	*Cicer arietinum*	** KT966800**	** KT966818**	**KT966836**	AY225398	NA
*Mesorhizobium *sp.	G-55	*Cicer arietinum*	** KT966801**	** KT966819**	**KT966837**	EU652149	NA
*Mesorhizobium *sp.	PII-3	*Cicer arietinum*	** KT966802**	** KT966820**	**KT966838**	EU652106	NA
*Mesorhizobium *sp.	PMI-6	*Cicer arietinum*	** KT966795**	** KT966821**	**KT966839**	EU652121	NA
*Mesorhizobium *sp.	S-1	*Cicer arietinum*	** KT966803**	** KT966822**	**KT966840**	EU652169	NA
*Mesorhizobium *sp.	S-8	*Cicer arietinum*	** KT966793**	** KT966823**	**KT966841**	EU652116	NA
*Mesorhizobium *sp.	SL-9	*Cicer arietinum*	** KT966804**	** KT966824**	**KT966842**	JN191663	NA
*Mesorhizobium *sp.	ST-2	*Cicer arietinum*	** KT966805**	** KT966825**	**KT966843**	AY225401	NA
*Mesorhizobium *sp.	STR-2	*Cicer arietinum*	** KT966806**	** KT966826**	**KT966844**	EU652117	NA
*Mesorhizobium *sp.	STR-14	*Cicer arietinum*	** KT966807**	** KT966827**	**KT966845**	EU652118	NA
*Mesorhizobium *sp.	V-15b	*Cicer arietinum*	** KT966808**	**KT966828**	**KT966846**	EF504315	NA

Bioproject ID was retrieved from JGI database [24] and NCBI accession numbers for sequences resulting from this study are shown in bold. NA, not available.

## Data Availability

The NCBI accession data that support the results are within the manuscript.
